# Evaluation Methods and Coupled Optimization at Macro- and Micro-Scales for Profiled Ring Rolling of Inconel718 Alloy

**DOI:** 10.3390/ma17184538

**Published:** 2024-09-15

**Authors:** Xinglin Zhu, Erting Dong, Xiaomin Qiao, Dong Liu

**Affiliations:** 1 School of Materials Science and Engineering, Henan Institute of Technology, Xinxiang 453000, China; donget@hait.edu.cn (E.D.); 13030326429@163.com (X.Q.); 2 Xinxiang Key Laboratory of Materials Processing Technology and Mould, Xinxiang 453000, China; 3 School of Materials Science and Engineering, Northwestern Polytechnical University, Xi’an 710072, China; liudong@nwpu.edu.cn

**Keywords:** profiled ring rolling, synthetic size factor, microstructure evolution model, finite element simulation, response surface methodology, Inconel718 alloy

## Abstract

The forming quality of profiled ring rolling not only encompasses macroscopic accuracy but also emphasizes the microstructure. Due to the multiple process parameters and complex metal flow during profiled ring rolling, the various forming defects are difficult to control and difficult to study theoretically. The objective of this study is to establish a comprehensive method for evaluating the forming quality of profiled rings, which considers both the macroscopic forming accuracy and the microstructure. Firstly, the synthetic size factor was defined, and the evolutionary relation between the section forming rate and the diameter growth rate of E-section ring rolling was analyzed in detail. The synthetic size factor can be used to describe the dimensional evolution and evaluate the forming accuracy of the profiled ring rolling process. Taking into full consideration the features of intermittent deformation in local areas, a microstructure evolution model of the Inconel718 alloy during E-section ring rolling, which can accurately predict the recrystallization volume fraction and average grain size of the final ring, was established. Then, combined with finite element simulation, the influence of the rotation speed of the driving roll on the macro-size evolution and microstructure was systematically analyzed. The results indicate that there is often a discrepancy between dimensional accuracy and microstructure uniformity in the optimization trend. For instance, the higher the rotation speed of the driving roll is, the more uniform the microstructure is, but the more difficult it is for the section profile to form. Finally, combined with response surface methodology (RSM), multi-parameter optimization was carried out with section forming accuracy and grain uniformity as the optimization objectives. By using the optimal parameters, an E-section ring with a complete profile and a uniform microstructure was obtained, with a maximum prediction error of the recrystallization volume fraction lower than 5%. The results show that the macroscopic and microscopic quality evaluation methods proposed in this study, as well as the optimization method combining RSM, can be effectively applied to the process optimization of profiled ring rolling.

## 1. Introduction

Profiled ring rolling is extensively employed in the production of various alloy rings for aircraft engines, including casings, flanges, and sealing rings, as it ensures a seamless integrated flow line and offers optimal material utilization [1,2]. Materials with good high-temperature performance, such as nickel-based superalloy and titanium alloy, are often used in aeroengine rings [3,4]. Forming accuracy and microstructure homogeneity are difficult to control in profiled ring rolling due to the complex ring profile and material nonlinearity. If the motion of each roll or the process parameters are not correctly set, various dimensional defects, such as incomplete sections, diameter errors, concentric deviations, edge burrs and local folds, are easily produced [5,6,7,8]. 

In recent years, research on hot ring rolling has focused on key issues, such as the control of the process parameters, blank design, geometric accuracy improvement and microstructure optimization [9,10]. Focusing on process parameter control, Gontarz et al. [11] analyzed the influence of the feed speed of the main roll and the preform temperature on the dimensional accuracy of a C45 steel ring. Guo et al. [12,13] analyzed the influences of the radii of the driving roll and mandrel and the feed rate per revolution on section forming quality, as well as strain and temperature distributions during profiled ring rolling. In relation to the control of rolling defects, Oh et al. [14,15] examined a commonly used L-shaped ring and explored the primary factors contributing to defects like incomplete filling and folding. They then aimed to minimize these defects through the optimization of key parameters, such as the feed rate of the mandrel and the rotation speed of the driving roll. Professor Allwood [16] proposed a new method of axial and circumferential constrained rolling to solve the problem of the forming accuracy of profiled rings. Focusing on the inner surface forming of the profiled ring, Han et al. [17] proposed a method of constrained rolling by using the ring envelope die, which restricted the circumferential flow of the metal and caused the metal to flow along the radial direction, thus forming the profiled structure on the inner surface.

The microstructure of the formed ring determines the final mechanical properties and service life. Research on the microstructure of the ring rolling process has been progressively advanced in recent years. Zhou et al. [18] analyzed the influence of the hot ring rolling process on the microstructural evolution of 100Cr6 bearing rings. According to the deformation characteristics of the material at high temperatures, Liu et al. [19,20] systematically analyzed the microstructure evolution and control method of a superalloy in the ring rolling process. Tang et al. [21] presented an internal state variable material model, which enables the unified prediction of flow behavior and microstructure evolution in the dynamic and post-dynamic regimes. Wang et al. [22] discussed the evolution of grain size and δ phase volume fraction during the hot ring rolling process of Ti-6Al-4V and explored the final microstructures under different processing conditions. 

Previous research played a crucial role in advancing the progress of profiled ring rolling techniques. However, with the increasing demand for intricate ring structures and ultra-large rings, particularly for materials such as titanium alloys and superalloys that are sensitive to the microstructure, there is now a greater emphasis on achieving enhanced process stability, precision in forming, and overall mechanical properties of the ring components [23,24,25,26]. Different from singular-compression deformation, the metal undergoes continuous and intermittent plastic deformation in the process of ring rolling. The local deformations of the blank occur in both the radial and axial directions within the rolling cavity between the driving roll and the mandrel, as well as between the conical rolls. The local metal undergoes alternating radial and axial deformations with the rotation of the ring. Especially for profiled rings, the stress state is more complex, and the strain rate, deformation temperature and effective strain in each deformation phase are different. Therefore, the stable and uniform microstructure of profiled rings is extremely challenging to achieve during the ring rolling process for the Inconel718 alloy, which exhibits a high degree of structural sensitivity. It is not sufficiently accurate to predict the final microstructure as a single-pass deformation process.

Therefore, based on a stable profiled ring rolling process, how to avoid different defects, reduce processing time, improve the material utilization rate and obtain complete profiled rings with a satisfactory microstructure have become the key problems at present [27,28]. At present, the theoretical analysis method and process optimization strategy of profiled ring rolling need to be further strengthened, and there are few methods that can be used to evaluate the forming quality of profiled rings from both the macroscopic size and the microscopic structure at the same time. In addition, limited by the calculation conditions and homogenization theory, the main problem at present is the weak coupling between scales. That is, the macro-model information cannot be accurately transmitted to the microstructure evolution model, and the microstructure information cannot be truly fed back to the macro finite element model [29,30]. Therefore, the development of multi-scale fully coupled numerical simulation of the ring rolling process in macro–micro scales is a crucial direction to pursue. The key to solving this problem is how to establish an information feedback channel between the macro-scale and micro-scale, based on a deep analysis of process deformation characteristics. The information that this channel can feed back and transmit will determine the degree of coupling between the different scales.

**Figure 1:**
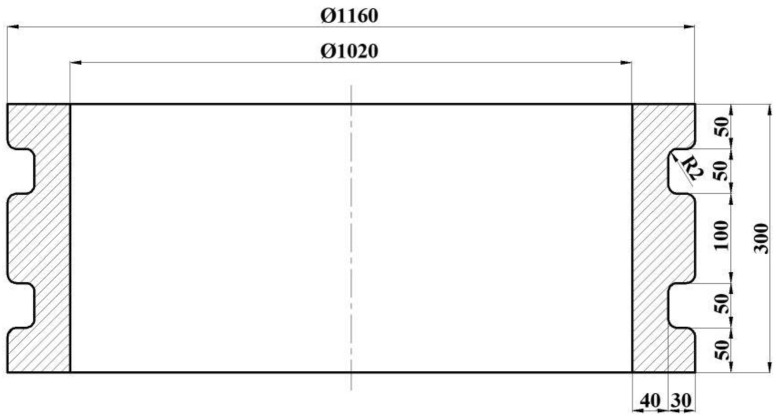
Diagram of the E-section ring.

In this study, a typical E-section ring, as shown in Figure 1, was chosen. Based on the forming characteristics of the profiled ring rolling process, a synthetic size factor was established to evaluate the macroscopic size accuracy, and a microstructure evolution model was developed to predict the microstructure distribution. By combining the finite element method (FEM) and the response surface methodology (RSM), the influences of key factors on forming quality and the methods for obtaining profiled rings that meet production requirements in terms of both macroscopic sizes and microscopic structure were thoroughly discussed. We confirm that this study is our original work and has not been copied from any other source without proper citation. We understand that this declaration is binding and will be considered in evaluating the originality of this study.

## 2. Finite Element Model

### 2.1. Establishment of FE Model

**Figure 2:**
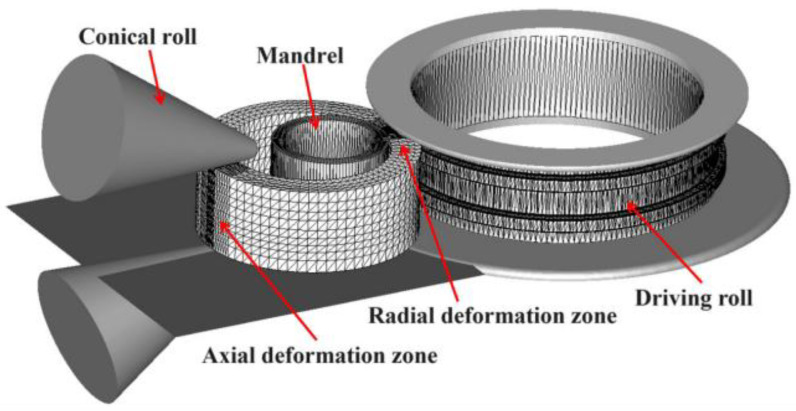
FE model of the E-section ring rolling process.

Based on the FORGE finite element software developed by Transvalor https://www.transvalor.com/en/forge (accessed on 3 September 2024), a 3D finite element model, as shown in Figure 2, was developed to simulate the E-section ring rolling process. Stress–strain curves of the Inconel718 alloy were obtained through isothermal compression tests at strain rates ranging from 0.001 s^−1^ to 10 s^−1^ and temperatures ranging from 940 °C to 1060 °C. Subsequently, the processed experimental data were incorporated into the FPD base of the FORGE software. 

To improve computational efficiency and to minimize the influence of ovality on the research results, the automatic centering function of the FORGE software was utilized, and the action of the guide roll was omitted. The motion state of each roll was controlled jointly by secondary development modules established in our previous research [31]. To ensure the stability and accuracy of the simulation process, the rotation speeds of the mandrel and the conical rolls were controlled by the iterative calculation module and the matching calculation module, respectively.

As shown in Figure 2, the blank was first sliced into different angles along the circumference. Then, a structured mesh was generated using tetrahedral elements of different sizes. The two deformation zones underwent radial deformation and slight axial deformation, respectively, during the ring rolling process. These zones were meshed into small elements in slices of 1 degree each and 2 degrees each to enhance computational accuracy. Other zones without deformation were meshed into larger elements in slices of 5 degrees each to conserve computational resources. The constant mesh size was set at 30 mm, and the volume size factor was set at 1.2. This structured mesh did not rotate with the ring’s rotation; instead, it maintained a relatively static spatial position. Additionally, a reasonable remeshing period was established to mitigate the influence of mesh distortion.

For the boundary conditions between the ring and the dies, the friction due to thermal deformation without lubrication and the thermal exchange of metal-hot were initially selected in the FORGE software. To improve simulation accuracy, the thermal exchange coefficient and friction factor for different contact states between the ring and rolls were measured using a ring upsetting experiment. Subsequently, the experimental data were used to refine these boundary conditions.

### 2.2. Experimental Verification

**Figure 3:**
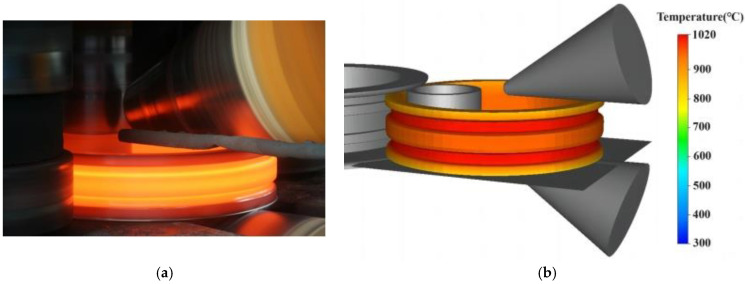
Comparison of the ring rolling processes. (**a**) Experiment; (**b**) simulation.

In order to validate the reliability of the FE model, an E-section ring rolling test of Inconel 718 alloy was conducted. The initial grain size of the blank is approximately 50 μm, the initial temperature is 1020 °C, the rotation speed of the driving roll is 16 r/min, and the feed speed of the mandrel is 1 mm/s. The rolling time is 65 s, and the feed speed of the mandrel decreases rapidly in the last 5 s to achieve rounding. During the experiment, the diameters of the ring were recorded in real time, and the rolling mill was equipped with a force sensor to measure the rolling forces in the feed direction. Subsequently, this ring rolling process was simulated with the same process parameters using the FE model established in this study. The experimental and simulated E-section ring rolling processes are compared in Figure 3.

**Figure 4:**
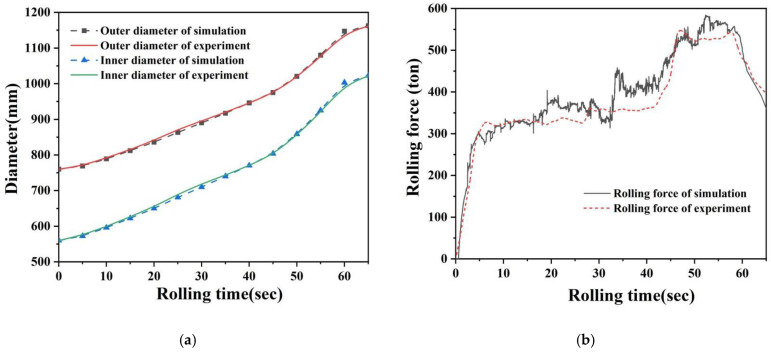
Comparison of experimental and simulation results. (**a**) Diameters; (**b**) rolling force.

A comparison between the simulation and experimental results is shown in Figure 4. As illustrated in Figure 4a, the evolution of the outer and inner diameters of the ring is consistent. In the experiment, the diameters grow slightly faster in the early stage than in the simulation, and they eventually reach constant sizes. The maximum difference in diameter is 8.5 mm, which could be caused by errors in the filling speed of the ring profile, inaccuracies in controlling equipment parameters, deviations in the roundness of the ring, or measurement errors. As shown in Figure 4b, as the ring profile gradually forms, the contact area between the blank and the rolls continues to increase, resulting in an increase in rolling force. Although the initial points of rapid increase in rolling force are slightly different between the experimental and simulated rolling processes, the development trend of the rolling force is consistent, and the error of the maximum rolling force is approximately 40 tons. This could be caused by inherent simulation errors, as well as fluctuations in the element sizes and contact conditions within the simulation. In general, the FE model for E-section ring rolling of the Inconel718 alloy developed in this study effectively replicates the actual production process, making it a reliable research tool that shortens the research period and reduces costs.

## 3. Evaluation on Forming Quality of Profiled Ring

### 3.1. Evaluation on Macroscopic Dimensional Accuracy

The profiled ring rolling process involves a co-evolution of cavity filling and ring diameter growth, but these rates do not always coincide. When the diameter grows at a higher rate than the cavity filling, the ring diameter may reach the desired size first, while the cavity cannot be fully filled at that time. If the rolling process continues, the diameter of the ring could exceed the dimensional tolerance. Conversely, if the diameter is significantly below the required value when the cavity is fully filled, the formed section may gradually disappear due to the circumferential flow of the metal during continuous rolling. Therefore, in the design of the rolling process, it is essential to ensure that the cavity filling rate and diameter growth rate are essentially the same, so that the desired size can be achieved simultaneously.

During the rolling process, the cross-sectional area of the rolling cavity gradually decreases as the mandrel feeds radially, causing the section of the blank to become consistent with the section of the cavity. Therefore, the ratio of the instantaneous cross-sectional area of the blank to that of the cavity (*S_t_*/*S_t_′*) is used to describe the progress of cavity filling. Similarly, the ratio of the instantaneous average diameter of the blank to the average diameter of the final ring (*d_t_*/*d*) is used to describe the diameter growth. After normalizing these two geometric parameters, the following results can be obtained:

(1) $$k_{F}=(\frac{S_{t}}{S_{t}\prime }-\frac{S_{0}}{S_{0}\prime })/(1-\frac{S_{0}}{S_{0}\prime }),$$

(2) $$k_{D}=(\frac{d_{t}}{d}-\frac{d_{0}}{d})/(1-\frac{d_{0}}{d}).$$

where *S_t_*, *S_t_*′ are the instantaneous cross-sectional areas of the blank and cavity, respectively. *S*_0_, *S*_0_′ are the initial cross-sectional areas of the blank and cavity, respectively. *d*_0_, *d_t_* and *d* are the average diameters of the initial blank, the instantaneous ring, and the final ring, respectively. *k_F_* and *k_D_* are the cavity filling rate and diameter growth rate, respectively. When *k_F_* and *k_D_* approach 1, the section forming tends to be complete, and the diameter of the blank approaches the required size.

To conveniently describe the relationship between the diameter growth rate and the cavity filling rate in the profiled ring rolling process, a synthetic size factor, denoted as *μ,* is defined as follows:

(3) $$\mu =k_{D}/k_{F}.$$

The synthetic size factor, denoted as *μ,* holds significant physical implications for describing the relationship between ring diameter growth and section shape formation during the profiled ring rolling process. Generally, the process of cavity filling precedes diameter growth, resulting in a small initial value for *μ*. To ensure the continuity of the curve for *μ* and to express the rolling process more clearly, when *t* = 0, *k_F_* and *k_D_* are 0, and, in this case, *μ* is defined as 0. 

**Figure 5:**
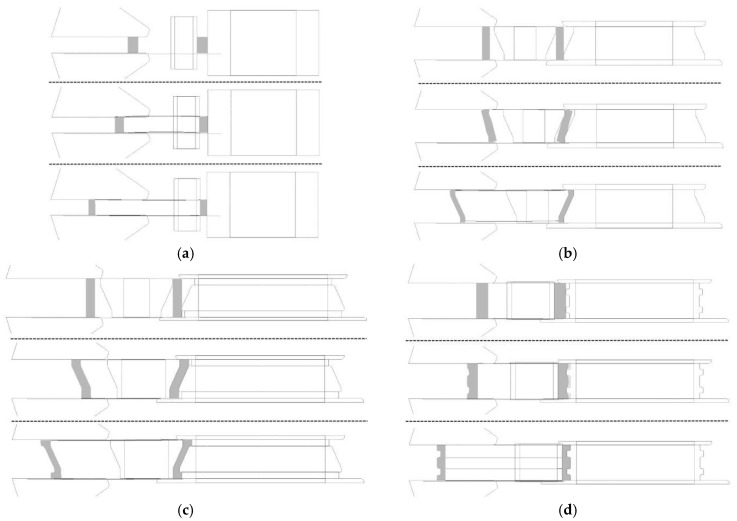
Ring rolling processes for different rings. (**a**) Rectangular ring; (**b**) I-section ring; (**c**) C-section ring; (**d**) E-section ring.

**Figure 6:**
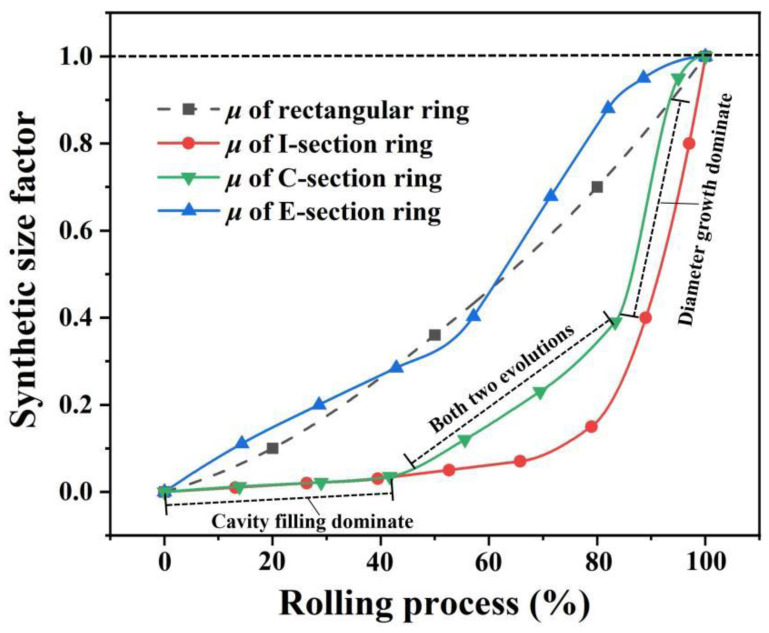
Synthetic size factor curves for different ring rolling processes.

To provide a clearer explanation of the synthetic size factor, the rolling processes of four different ring types, as depicted in Figure 5, were selected for analysis of the evolution of ring size during the ring rolling process. According to Equations (1) to (3), the synthetic size factor curves for these four different ring rolling processes were calculated and are shown in Figure 6. Different from the profiled rings, the rolling cavity of the rectangular ring is consistently filled, and *k_F_* is always equal to 1. Therefore, the synthetic size factor curve represents the growth rate of the ring diameter. When the feed rate is constant, *μ* increases in an approximately linear manner. At the early stage of inclined I-section ring rolling, the taper of the ring forms rapidly, but the diameter remains essentially unchanged, resulting in a very small slope in the synthetic size factor curve at this stage. When the section is essentially formed, the diameter of the ring grows rapidly as the mandrel continues to feed, which is reflected as a rapid increase in the slope of the curve. During the C-section ring rolling process, the taper of the ring is also formed in the early stage, when cavity filling is dominant, resulting in a very small slope of the curve. In the middle stage, after the taper has formed, the two steps of the C-section ring begin to fill in, and the diameters start growing faster. In the final stage, the ring section is essentially formed, the diameter growth becomes dominant, and the slope of the corresponding curve is very large. Unlike the C-section ring, the E-section ring does not have a taper, so the diameter grows at a faster rate in the initial stage, and the growth rate also changes as the step is filled. 

In general, the lower the slope of the synthetic size factor curve, the faster the cavity fills and the slower the diameter grows. In practical production, it is expected that *μ* remains less than or equal to 1, indicating that the degree of cavity filling should always slightly exceed or match the degree of diameter growth. The cavity is filled first, with *μ* reaching 1 at the end of rolling, ensuring complete filling when the ring diameter meets the requirements. 

### 3.2. Evaluation on Microstructure

**Figure 7:**
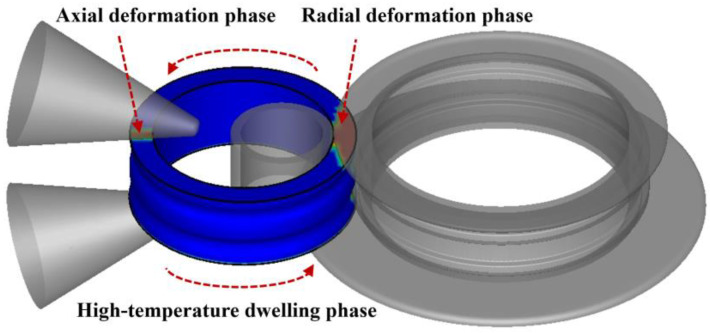
Diagram of the different phases in profiled ring rolling.

In order to accurately predict the microstructure evolution of the Inconel718 alloy during the ring rolling process, as shown in Figure 7, one rotation of the material was divided into four phases: the radial deformation phase, the axial deformation phase and two high-temperature dwelling phases. During the deformation phases, dynamic recrystallization occurs when the deformation exceeds the critical strain. The high-temperature dwelling phases do not provide a sufficient incubation time for static recrystallization, but meta-dynamic recrystallization occurs. Therefore, the evolution of the microstructure is a process in which dynamic and meta-dynamic processes alternate. The successive phases must consider the historical microstructure upon the conclusion of the preceding phase. The data necessary for the microstructure evolution model were exclusively derived from the FE model established in this study. By organically integrating the microstructure evolution model with the finite element model, precise predictions of the microstructure in profiled ring rolling can be achieved, providing technical support for process optimization.

**Figure 8:**
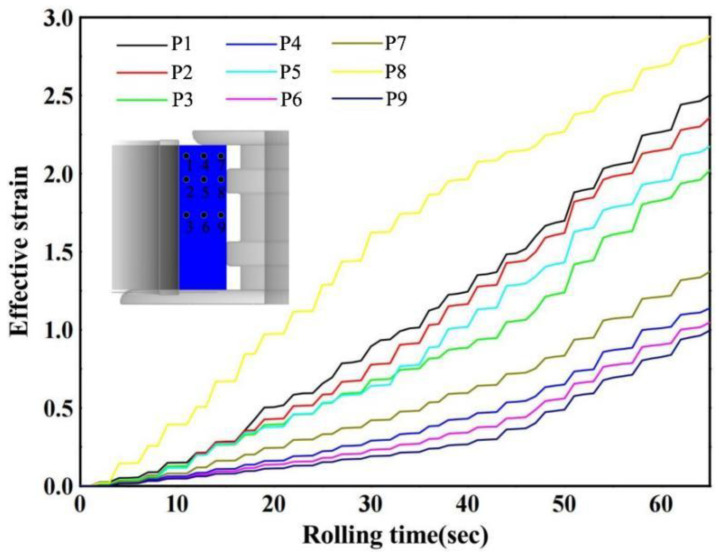
Strain evolution at nine tracking points.

According to the process parameters of the verification experiment in Section 2, the microstructure evolution process of the E-section ring rolling was simulated. Based on the structural characteristics of the E-section ring, nine tracking points were selected, as shown in Figure 8. It can be seen in Figure 8 that the effective strain exhibits a gradual increase. The radial deformation of the material was not significant, due to minimal changes in the height of the ring, whereas a noticeable strain increment occurs when the metal passes between the driving roll and the mandrel.

In the microstructure evolution model, the grains at each tracking point are divided into three distinct grain types: dynamic recrystallization grains, meta-dynamic recrystallization grains, and non-recrystallization grains. Each type is calculated separately. After undergoing a recrystallization process, the recrystallized grains are categorized into the corresponding recrystallization type and are separated from those that have not recrystallized. For each individual dynamic and meta-dynamic process, the calculation of microstructure evolution employs the mathematical models for recrystallization volume fraction and grain size, as established in our previous research, which is detailed in reference [32].

**Figure 9:**
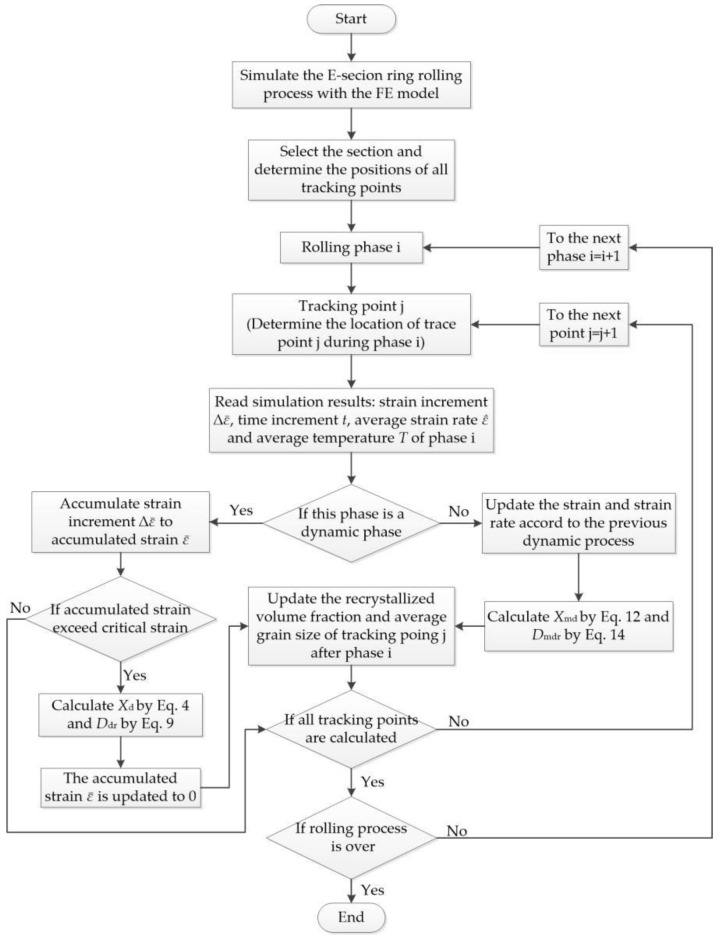
Flow chart of the microstructure evolution model for profiled ring rolling.

The grain size and volume fraction of dynamic recrystallization were quantitatively determined using a thermal simulation compression experiment on the Inconel718 alloy. Homogenized cylindrical samples with a diameter of 8 mm and a length of 10 mm were used in the experiment. The experimental temperatures were 960 °C, 980 °C, 1000 °C, and 1020 °C. The strain rates were 50 s^−1^, 10 s^−1^, 1 s^−1^, 0.1 s^−1^, and 0.01 s^−1^. The true strains were 0.357, 0.693, and 0.916. The high-temperature dwelling times after deformation were 0 s, 5 s, 10 s, and 15 s. After deformation and insulation, the microstructure at high temperature was retained by rapid cooling. Following the metallographic test, the recrystallization volume fraction and grain size were quantitatively measured using an automatic image analyzer. Subsequently, mathematical models of microstructure evolution were established based on the analysis of experimental data. When these mathematical models were integrated into the microstructure evolution model, the deformation characteristics of profiled ring rolling were fully considered. Figure 9 shows a flow chart of the calculation process for the microstructure evolution model, and the key points in the calculation process are explained as follows.

(1) When a tracking point undergoes a deformation phase, the strain increment  is accumulated into the variable $\bar{\varepsilon }$. When the cumulative strain exceeds the critical strain ($\bar{\varepsilon }>{\bar{\varepsilon }}_{c}$), dynamic recrystallization occurs, and the recrystallization volume fraction $X_{d}$ is calculated from Equation (4).

(4) $$X_{d}=1-\exp{\left[-0.693\times (\frac{\bar{\varepsilon }-{\bar{\varepsilon }}_{c}}{{\bar{\varepsilon }}_{0.5}}\right)}^{1.15}].$$

where $\bar{\varepsilon }$ is the effective strain; ${\bar{\varepsilon }}_{c}$, ${\bar{\varepsilon }}_{0.5}$ are the critical effective strain and the effective strain leading to *X_d_* = 0.5, which can be determined by Equation (5) and Equation (6), respectively.

(5) $${\bar{\varepsilon }}_{c}=\beta \times {\bar{\varepsilon }}_{p}\;(\beta =0.8\sim 0.9).$$

(6) $${\bar{\varepsilon }}_{0.5}=0.0019\times Z^{0.126}.$$

where ${\bar{\varepsilon }}_{p}$ is the peak strain determined by Equation (7), and *Z* is the Zener–Hollomon parameter determined by Equation (8).

(7) $$\ln{\bar{\varepsilon }}_{p}=0.0048{\left(\ln Z\right)}^{3}-0.5349{\left(\ln Z\right)}^{2}+19.66(\ln Z)-242.36.$$

(8) $$Z=\dot{\bar{\varepsilon }}-\exp[Q/(RT)].$$

where $\dot{\bar{\varepsilon }}$ is the average strain rate, *Q* is the activation energy of deformation, *R* is the gas constant, and *T* is the temperature.

(2) According to the experiment, the dynamic recrystallized grains promptly attain a certain size after nucleation, and this size is contingent upon both temperature and strain rate, irrespective of the original grain size. During the subsequent deformation process, the size of the recrystallization grains does not change significantly. Based on the aforementioned facts, the dynamic recrystallization grain size *D_dr_* can be calculated using Equation (9).

(9) $$\ln D_{dr}=F_{1}+F_{2}\times \frac{T-T_{p}}{1000}.$$

where *T* and *T_p_* are the deformation temperature and the dissolution temperature of the delta phase, respectively. *F*_1_ and *F*_2_ are functions of the strain rate, which can be determined by Equation (10) and Equation (11), respectively.

(10) $$F_{1}=9.685{\left(\frac{\ln\dot{\bar{\varepsilon }}}{10}\right)}^{4}-5.296{\left(\frac{\ln\dot{\bar{\varepsilon }}}{10}\right)}^{3}-1.342{\left(\frac{\ln\dot{\bar{\varepsilon }}}{10}\right)}^{2}+1.61(\frac{\ln\dot{\bar{\varepsilon }}}{10})+1.361.$$

(11) $$F_{2}=0.0261{\left(\ln\dot{\bar{\varepsilon }}\right)}^{4}+0.0976{\left(\ln\dot{\bar{\varepsilon }}\right)}^{3}-0.593{\left(\ln\dot{\bar{\varepsilon }}\right)}^{2}-1.51\ln\dot{\bar{\varepsilon }}+10.26.$$

(3) During the high-temperature dwelling phase, the strain rate $\dot{\bar{\varepsilon }}$ and effective strain $\bar{\varepsilon }$ of the meta-dynamic process are determined by the preceding dynamic process, and the average temperature of the high-temperature dwelling phase is selected. Then, the meta-dynamic recrystallization volume fraction *X_md_* can be calculated using Equation (12).

(12) $$X_{md}=1-\exp[-0.693\exp{\left(\frac{t}{t_{0.5}}\right)}^{2}].$$

where *t* is the high-temperature dwelling time, and *t*_0.5_ is the dwelling time that leads to *X_md_* = 0.5, which is determined by Equation (13).

(13) $$t_{0.5}=1.346\times 10^{-3}\times {\dot{\bar{\varepsilon }}}^{-0.86}\times {\bar{\varepsilon }}^{-1.36}\times \exp(\frac{8875}{T}).$$

(4) The experimental results show that grains generated by meta-dynamic recrystallization are generally larger than those generated by dynamic recrystallization. The meta-dynamic recrystallization grain size *D_mdr_* can be calculated using Equation (13).

(14) $$D_{mdr}=0.00538\times {\bar{\varepsilon }}^{\left(9.56-0.00803T\right)}\times {\dot{\bar{\varepsilon }}}^{\left(0.029T-0.0000117T^{2}-18.13\right)}\times \exp(0.00549T).$$

(5) After undergoing recrystallization, whether dynamic or subdynamic, the volume fraction of the recrystallized grains is incorporated into the corresponding grain type at the tracking point, while the strain accumulation variable is reset to zero. If no recrystallization occurs at this point during a particular phase, strain continues to accumulate. The average grain size at each tracking point is determined through a weighted summation of the recrystallization volume fraction and the grain size generated at each stage.

Since the grains tend to be uniform and equiaxial after holding above the delta phase transition temperature, the influence of the initial grain size on the recrystallization process is not significant. Therefore, the microstructure model adopted ignores the influence of the initial grain size. The composition of the material, particularly the niobium element content, exerts a noticeable influence on the recrystallization process. However, this study primarily focuses on establishing a microstructure prediction method for profiled ring rolling, thus disregarding the impact of material composition. By modifying some experimental data, this model can be directly applied to the ring rolling of similar nickel-based superalloys. Additionally, the treatment method for intermittent local deformation and the computational approach to microstructure evolution can be applied to other alloy materials.

## 4. Influence of Rotation Speed of Driving Roll

Numerous parameters influence the evolution of ring sizes in the profiled ring rolling process. When the feed speed of the mandrel is determined, the rotation speed of the driving roll emerges as a crucial parameter affecting the disparity in wall thickness between passive and active deformation regions. A higher rotation speed of the driving roll results in smaller reductions in wall thickness per revolution. Therefore, taking into account synthetic size factors, this study analyzes how key parameters, such as the rotation speed of the driving roll, impact the forming process.

With an initial blank temperature of 1020 °C, an FE model was utilized to simulate the forming processes with different rotation speeds (8 r/min, 12 r/min, 16 r/min, and 20 r/min) of the driving roll at a mandrel feed speed of 1 mm/s. Subsequently, the obtained FE data were applied to the microstructure evolution model. With the simulation results, the influence of the rotation speed on both macroscopic dimensional accuracy and microstructure were analyzed in detail.

### 4.1. Influence on Macroscopic Dimensional Accuracy

**Figure 10:**
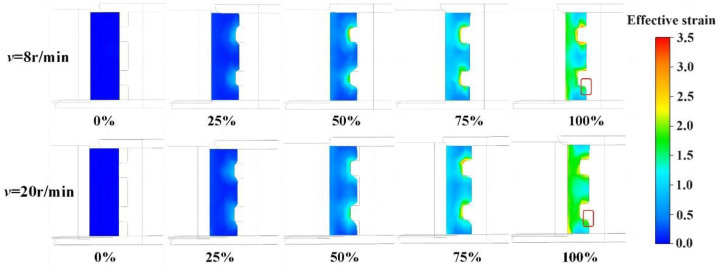
Section forming process at rotation speeds of 8 r/min and 20 r/min.

The simulation results of the section forming process with rotation speeds of 8 r/min and 20 r/min are presented in Figure 10. It can be observed that a lower rotation speed of the driving roll leads to less circumferential flow of the metal, resulting in a reduced thinning rate in the passive deformation region and an improved cavity filling rate. Conversely, a higher rotation speed induces a greater tangential force on the blank, causing increased circumferential flow of the metal, which leads to slower cavity filling and faster diameter growth. Although it is difficult to obtain a complete section with a high rotation speed, the strain distribution is more uniform. 

**Figure 11:**
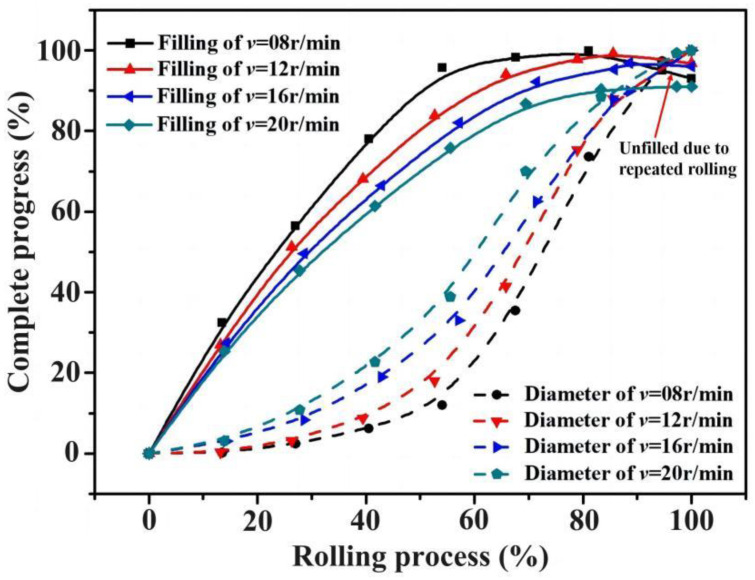
Process curves for cavity filling and diameter growth based on FE data.

To facilitate a more intuitive comparison, the process data for section area and diameter were extracted from the simulation results during the forming process. The process curves for cavity filling and diameter growth at different rotation speeds of the driving roll were calculated according to Equation (1) and Equation (2), respectively. These curves are presented in Figure 11, revealing that higher rotation speeds of the driving roll correspond to lower cavity filling rates but higher diameter growth rates. For instance, at a rotation speed of 20 r/min, when the process reaches 80% completion, the diameter growth rate surpasses the cavity filling rate. This ultimately results in incomplete cavity filling once the ring reaches its final diameter. 

On the other hand, at a rotational speed of 8 r/min, when the cavity is approximately 98% filled, the diameter increases by only about 75%. Continued rolling may lead to the loss of the formed section due to circumferential metal flow, resulting in the “repeated rolling” phenomenon. Furthermore, according to the laws of metal flow, when the disparity between the cavity filling rate and the diameter growth rate is too great, the metal is more susceptible to local deformation, which is detrimental to the uniformity of strain distribution. 

**Figure 12:**
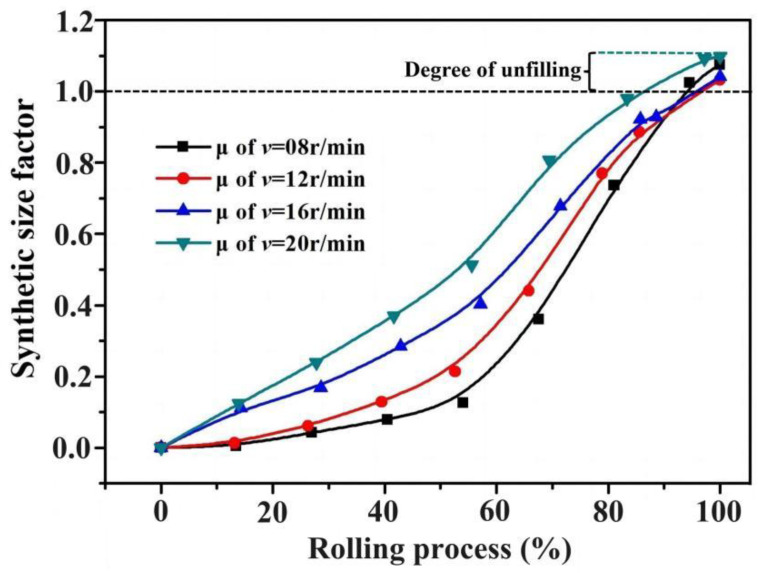
Synthetic size factor curves for different rotation speeds based on FE data.

Figure 12 shows the synthetic size factor curves for different rotation speeds of the driving roll. It can be observed that the curves have small slopes during the initial stage of rolling, indicating that section forming is the primary deformation mechanism, and the diameter grows slowly. In the later stage, the curves exhibit a rapid rise, which means that the diameters begin to grow rapidly. If the synthetic size factor exceeds 1 at the end of rolling, it indicates that the cavity cannot be filled completely, and the larger the synthetic size factor, the more incomplete the formed section will be.

According to the above analysis, dimensional accuracy and strain distribution should be carefully considered for different production requirements. Multiple parameters need to be optimized simultaneously, and the most optimal combination of these parameters should be studied to achieve the best-quality ring.

### 4.2. Influence on Microstructure

**Figure 13:**
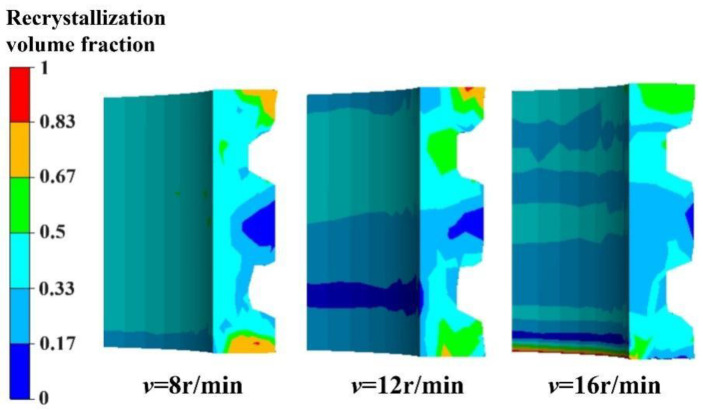
Recrystallization volume fractions at different rotation speeds of the driving roll.

**Figure 14:**
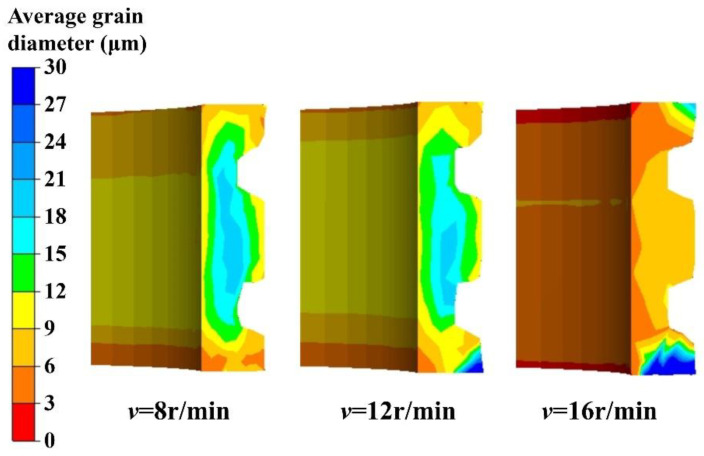
Average grain diameters at different rotation speeds of the driving roll.

The simulation results for the recrystallization volume fraction and the average grain diameter at different rotation speeds of the driving roll are presented in Figure 13 and Figure 14, respectively. The results indicate that the higher the rotation speed of the driving roll, the smaller the recrystallization volume fraction and the larger the average grain size at the two ends of the E-section ring. However, as the rotation speed increases, there is a noticeable increase in the recrystallization volume fraction in the middle part of the ring. Additionally, the average grain size decreases significantly, and the distribution of the recrystallization volume fraction becomes more uniform. This is because, with an increase in the rotation speed of the driving roll, the metal tends to flow circumferentially rather than radially to fill the rolling cavity. This results in insufficient deformation and incomplete recrystallization at the step of the ring section, leading to a larger average grain diameter in this area. In the middle part of the ring, the recrystallization degree is improved, and the average grain size is smaller due to the higher deformation rate.

It can be deduced that as the rotation speed of the driving roll increases, the distribution of the recrystallization volume fraction becomes more uniform, and the area with larger grain distribution shifts from the middle part to both ends of the ring.

## 5. Multi-Parameter Optimization Based on RSM

### 5.1. Experiment Design

Design variables: The rotation speed of the driving roll, which determines the linear velocity at the contact area between the driving roll, mandrel, and conical roll, is selected as the first design variable *a*. The radial feed rate of the mandrel, which causes the radial deformation of the ring and determines the rolling time, is selected as the second design variable *b*. The rolling temperature, which influences the material’s deformation resistance and thereby affects metal flow and strain distribution, is selected as the third design variable *c*.

Objective function: In this study, both the forming accuracy and microstructure distribution of the ring were considered. Consequently, the cavity filling rate and microstructure uniformity at the end were taken as the two responses, determined by Equation (15) and Equation (16), respectively.

(15) $$\theta_{1}=1-k_{F}=1-(\frac{S_{t}}{S_{t}^{\prime }}-\frac{S_{0}}{S_{0}^{\prime }})/(1-\frac{S_{0}}{S_{0}^{\prime }}).$$

When *k_F_* is close to 1, the smaller *θ*_1_ *is*, the more complete the ring section will be.

(16) $$\theta_{2}=S_{d}=\sqrt{\frac{1}{n}\sum_{i=1}^{n}(d_{i}-\bar{d}{)}^{2}}$$

where *d_i_* and *d*, respectively, represent the average grain diameter of tracking point *i* and the average grain diameter of all tracking points. The smaller *θ*_2_ is, the more uniform the microstructure will be.

The values of *θ*_1_ and *θ*_2_ obtained from the simulation were mapped to the range [0–1] using Min–Max Normalization to make their differences more significant. The transformed response values are *ϕ*_1_ and *ϕ*_2_*,* respectively. Then, the objective function can be defined as follows:

(17) $$\left\{ \begin{array}{ll} \mathrm{Variables}\; & a,b,c\\ \mathrm{Min}\; & \phi (a,b,c)=0.4\varphi_{1}+0.6\varphi_{2} \end{array}.\right.$$

A Center Composite Design (CCD) was utilized to design the experiment. As shown in Table 1, a three-factor, five-level design was adopted, and the values for each level were standardized using Equation (18) to normalize the variables. Subsequently, twenty groups of experimental combinations were developed, as presented in Table 2, which included five repeated experiments. The simulation results, after normalization, are also listed in Table 2.

(18) $$\delta_{i}=\frac{2X-(X_{\max}+X_{\min})}{(X_{\max}-X_{\min})/2}.$$

where *δ_i_* represents the coded value of the target variable *X* for the design variable. *X*_max_ and *X*_min_ are the maximum and minimum values of the corresponding design variable, respectively.

### 5.2. Result Analysis

**Figure 15:**
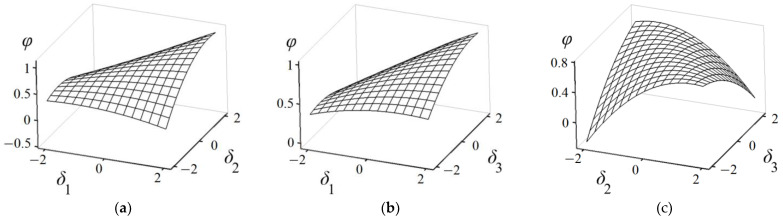
Three-dimensional surface graphs of variable–response relationships. (**a**) Regarding *δ*_1_ and *δ*_2_; (**b**) regarding *δ*_1_ and *δ*_3_; (**c**) regarding *δ*_2_ and *δ*_3_.

According to the simulation data presented in Table 2, a second-order response model, as shown in Equation (19), was derived through regression analysis. The multivariate correlation coefficient *R*^2^ can be utilized to assess the fitting accuracy of the model. After accounting for the outliers, the fitting accuracy *R*^2^ of Equation (19) achieved 92.97%, indicating that the response relationship between the design variables and the objective function is effectively captured by this model. Figure 15 presents 3D surface graphs that illustrate the objective response with respect to different variables. When one variable is held constant, the influence of the other two variables on the objective function can be visually analyzed using the 3D surface graph.

(19) $$\begin{array}{l} f=0.6890+0.1465\delta_{1}+0.0277\delta_{2}+0.0134\delta_{3}-0.0203{\delta_{1}}^{2}-0.0756{\delta_{2}}^{2}\\ -0.0368{\delta_{3}}^{2}+0.0958\delta_{1}\delta_{2}+0.0511\delta_{1}\delta_{3}-0.1096\delta_{2}\delta_{3}. \end{array}$$

**Figure 16:**
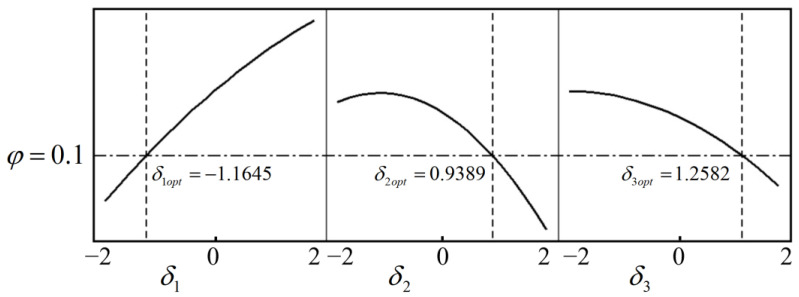
Optimization chart for a target response of 0.1.

According to the response model, response curves for the three variables can be derived under various constraints for different target responses. For instance, when the target response is set to 0.1 and the rolling temperature is confined to a range of 1020 °C to 1060 °C, the optimization chart, as depicted in Figure 16, can be generated. Concurrently, the most optimal combination of coded variables can be determined as *δ*_1_ = −1.1645, *δ*_2_ = 0.9389, *δ*_3_ = 1.2582. Once these values are transformed using Equation (18), the corresponding design variable combination can be ascertained based on actual production conditions, yielding *a* = 11 r/min, *b* = 1.2 mm/s, *c* = 1045 °C. 

**Figure 17:**
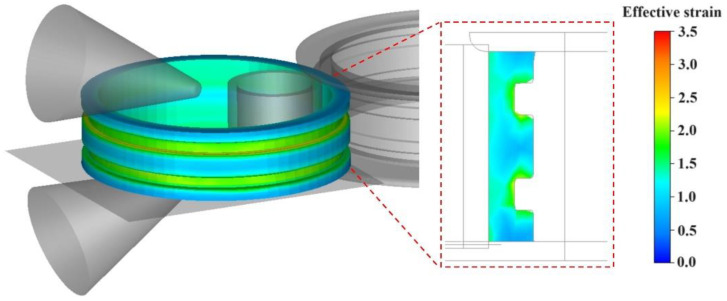
Strain distribution of the formed ring using the most optimal parameters.

The E-section ring rolling process was simulated using the most optimal parameters, and the simulation results, as presented in Figure 17, indicate a stable forming process and successful cavity filling. According to the strain distribution diagram, the maximum difference in effective strain is 0.8, which indicates that the deformation is uniform. 

**Figure 18:**
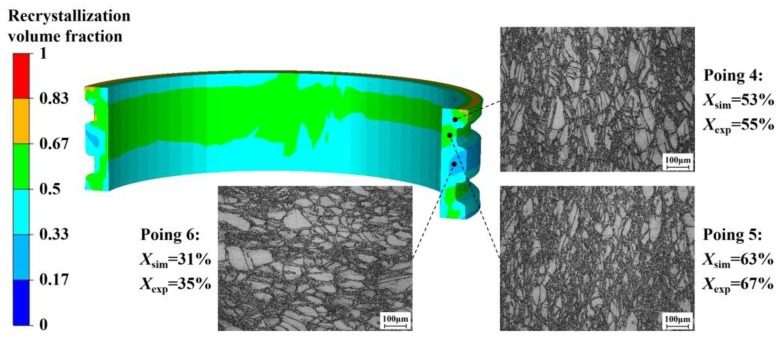
Simulation and experimental results for recrystallization volume fraction.

To verify the prediction accuracy of the finial microstructure of the E-section ring, three sampling points were selected: at the 1/2 thickness of the upper step, the groove, and the middle step, corresponding to tracking points 4, 5, and 6, as indicated in Figure 8. Subsequently, metallographic experiments were conducted to verify the predicted results. Figure 18 compares the simulation results with the experimental results for the recrystallization volume fraction using the most optimal parameters. The simulation results for the recrystallization volume fraction at tracking points 4, 5, and 6 are 53%, 63%, and 31%, respectively, while the experimental results of the corresponding locations are 55%, 67%, and 35%, respectively. The optimal simulation results are in close agreement with the experimental results, with a maximum absolute error of less than 5%. 

The experimental results indicate that the effective strain generated at the groove (tracking point 5) is the highest, where the recrystallization volume fraction is also the greatest. It can be observed that the recrystallized grains at tracking point 5 are finer, which suggests that the proportion of dynamically recrystallized grains is larger. This observation is consistent with the predicted microstructural results. The strains at tracking points 4 and 6 are comparatively minor, where recrystallized grains are predominantly formed through meta-dynamic recrystallization, resulting in relatively lower cumulative recrystallization volume fractions. Through the coupled optimization of process parameters, the average grain size at different points becomes more uniform, and an E-section ring with a more complete profile is obtained.

The synthetic size factor, as defined in this study, can directly and clearly depict the size evolution of the profiled ring during the rolling process and effectively evaluate its macroscopic dimensional accuracy. Additionally, the microstructure evolution model for profiled ring rolling developed in this study can accurately predict both the recrystallization process and the final grain size in the profiled ring rolling process. The results indicate that the multi-objective and multi-parameter coupled optimization method, which combines FEM and RSM, can be effectively applied in the actual production of profiled rings when using these evaluation methods.

## 6. Conclusions

In this study, with the establishment of a synthetic size factor and a microstructure evolution model, the prediction and evaluation of both the macro size and microstructure of the profiled ring rolling process were realized. Additionally, the multi-objective and multi-parameter coupled optimization was achieved by combining FEM and RSM.

(1) The synthetic size factor, *μ = k_D_*/*k_F_,* was defined to describe the evolution relationship between the diameter growth rate and the cavity filling rate during the profiled ring rolling process. This factor can be used to evaluate the macroscopic dimensional accuracy of the profiled ring.

(2) The microstructure evolution model for the Inconel718 alloy during the profiled ring rolling process was established based on the single deformation microstructure evolution model and FEM simulation data. This model fully considers the characteristics of local intermittent deformation and the inheritance of historical data at each stage, which enhances the prediction accuracy of dynamic recrystallization and enables prediction of meta-dynamic recrystallization during the high-temperature dwelling phase.

(3) Taking the E-section ring rolling as an example, the influence of the driving roll’s rotation speed on the forming quality was thoroughly discussed. If the cavity filling rate is lower than the diameter growth rate, the final section cannot be formed completely. However, premature formation of the section is not conducive to strain uniformity, and continued rolling may lead to the loss of the formed section due to the circumferential flow of the metal, resulting in the “repeated rolling” phenomenon. As the rotation speed of the driving roll increases, the distribution of the recrystallization volume fraction becomes more uniform, and the area with larger grain distribution shifts from the middle part to both ends of the ring.

(4) For the combined optimization of macroscopic dimensional accuracy and microstructure, a reliable method was implemented to obtain the most optimal parameter combination for E-section ring rolling under certain constraints through the combination of RSM and FEM. By using the optimal process parameters, an E-section ring with a complete profile was obtained. Meanwhile, three tracking locations with significant deformation differences were selected for observation. The recrystallization volume fractions at these locations, as observed through metallographic experiments, were 55%, 67%, and 35%, respectively. The experimental results exhibit good microstructure uniformity and align consistently with the simulation results, demonstrating a maximum absolute prediction error of less than 5%. The results demonstrate that the macroscopic and microscopic quality evaluation methods, as well as the optimization method that combines RSM, proposed in this study, can be effectively applied to the process optimization of profiled ring rolling.

In subsequent studies, achieving the feedback coupling of the interactions between macro size and microstructure and developing a multi-scale fully coupled numerical simulation of the profiled ring rolling process are crucial directions.

## Figures and Tables

**Table 1:** CCD factor levels for the E-section ring rolling.

Design Variable	Coded Symbol	Level (Coded)				
		−2	−1	0	1	2
*a* [r/min]	*δ*_1_	8	12	16	20	24
*b* [mm/s]	*δ*_2_	0.6	0.8	1	1.2	1.4
*c* [ °C]	*δ*_3_	980	1000	1020	1040	1060

**Table 2:** Design experiments and experimental results.

No.	*δ*_1_	*δ*_2_	*δ*_3_	*ϕ*_1_	*ϕ*_2_	*φ*
1	−1	−1	1	0.56	0.55	0.55
2	0	0	0	0.67	0.7	0.69
3	0	2	0	0.33	0.59	0.49
4	0	0	2	0.33	0.73	0.57
5	1	−1	−1	0.89	0	0.36
6	2	0	0	0.89	1	0.96
7	1	−1	1	1.00	0.68	0.81
8	0	0	0	0.67	0.7	0.69
9	0	0	−2	0.56	0.46	0.50
10	0	0	0	0.67	0.7	0.69
11	−1	−1	−1	0.44	0.59	0.53
12	0	0	0	0.67	0.7	0.69
13	1	1	1	0.44	0.82	0.67
14	1	1	−1	0.89	0.88	0.88
15	−1	1	−1	0.22	0.6	0.45
16	0	−2	0	0.50	0.12	0.27
17	−1	1	1	0.17	0.32	0.26
18	0	0	0	0.67	0.7	0.69
19	0	0	0	0.67	0.7	0.69
20	−2	0	0	0.00	0.41	0.25

## Data Availability

The original contributions presented in this study are included in the article; further inquiries can be directed to the corresponding author.
